# Artefact Profiling: Panomics Approaches for Understanding the Materiality of Written Artefacts

**DOI:** 10.3390/molecules28124872

**Published:** 2023-06-20

**Authors:** Marina Creydt, Markus Fischer

**Affiliations:** 1 Institute of Food Chemistry, Hamburg School of Food Science, University of Hamburg, Grindelallee 117, 20146 Hamburg, Germany; markus.fischer@uni-hamburg.de; 2 Cluster of Excellence, Understanding Written Artefacts, University of Hamburg, Warburgstraße 26, 20354 Hamburg, Germany

**Keywords:** ancient artefacts, written manuscripts, omics strategies, panomics, artefact profiling, archaeometry

## Abstract

This review explains the strategies behind genomics, proteomics, metabolomics, metallomics and isotopolomics approaches and their applicability to written artefacts. The respective sub-chapters give an insight into the analytical procedure and the conclusions drawn from such analyses. A distinction is made between information that can be obtained from the materials used in the respective manuscript and meta-information that cannot be obtained from the manuscript itself, but from residues of organisms such as bacteria or the authors and readers. In addition, various sampling techniques are discussed in particular, which pose a special challenge in manuscripts. The focus is on high-resolution, non-targeted strategies that can be used to extract the maximum amount of information about ancient objects. The combination of the various omics disciplines (panomics) especially offers potential added value in terms of the best possible interpretations of the data received. The information obtained can be used to understand the production of ancient artefacts, to gain impressions of former living conditions, to prove their authenticity, to assess whether there is a toxic hazard in handling the manuscripts, and to be able to determine appropriate measures for their conservation and restoration.

## 1. Introduction

In recent years, it has increasingly become apparent that cooperation between the humanities and natural sciences represents a particularly valuable symbiosis for the preservation of the common cultural heritage [1]. In this context, the so-called omics disciplines for the chemical analysis of historical artefacts have received enormous attention. However, its application to ancient objects is still in its infancy. Nevertheless, in manuscript research, the term “biocodicology” has taken on special significance, introduced only a few years ago by Fiddyment et al., referring to the study of biological information in a manuscript using genomics and proteomics strategies [2]. We propose to extend this approach to include further omics disciplines such as metabolomics and metallomics as well as isotopolomics, as these are also suitable for chemical analyses of ancient manuscripts and provide additional information.

While omics disciplines have been applied for several years in medicine, plant breeding, or food quality control, the application to ancient objects is relatively new [3,4]. Occasionally, the term paleo-omics is also used in this context [5]. Paleo-omics strategies have so far been applied mainly to understand biodeterioration processes and, if necessary, to adopt appropriate strategies for conservation. Therefore, the focus has been predominantly on the analysis of microorganisms. Thus, these are actually meta-omics methods since it is not the objects themselves that are detected, but residues from other organisms [6,7,8,9,10,11]. Nevertheless, the analysis of ancient materials using omics strategies has also increased in recent years. In the case of manuscripts, the focus is in particular on the writing surfaces and the inks, dyes, and pigments [2,12,13].

**Figure 1:**
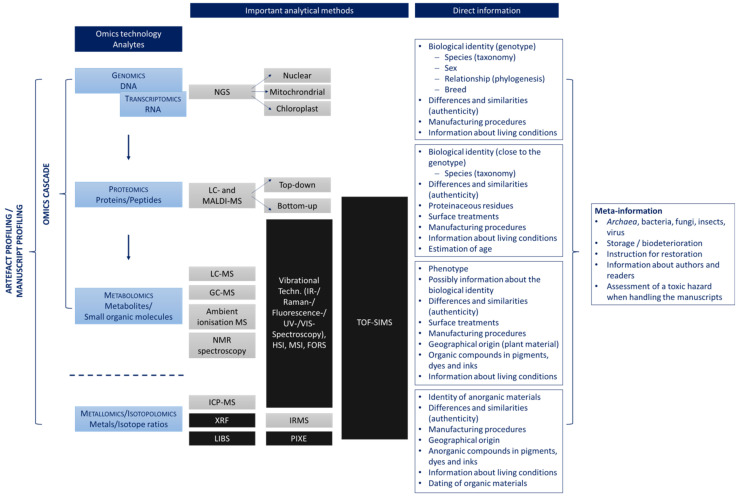
Omics disciplines that can be applied to the analysis of ancient artifacts including the analysis methods commonly used. High-resolution platforms typically applied for omics analysis are colored grey. Low-resolution methods are coloured black. They are also commonly applied for analysing ancient artefacts, but they do not offer the resolution and sensitivity required for omics approaches. In addition, it is shown for which scientific questions the different strategies can be used. While the direct information can be taken from the writing surfaces, inks, dyes, or pigments, meta-information results from residues from other organisms. Abbreviations: GC-MS, gas chromatography mass spectrometry; FORS, Fiber Optics Reflectance Spectroscopy; HIS, hyperspectral imaging; ICP-MS, inductively coupled plasma mass spectrometry; IR, infrared; IRMS, isotope ratio mass spectrometry; LC-MS, liquid chromatography mass spectrometry; LIBS, laser-induced breakdown spectroscopy; MALDI-MS, matrix-assisted laser desorption/ionisation mass spectrometry; MSI, multispectral imaging; NGS, next generation sequencing; NMR, nuclear magnetic resonance; PIXE, particle-induced X-ray emission; TOF-SIMS; time-of-flight secondary ion mass spectrometry; UV, ultraviolet; VIS, visible; XRF, X-ray fluorescence.

In general, the suffix -omics refers to comparative chemical analyses with which a complete or almost complete cellular level, tissue, organ, or organism can be recorded. However, in this regard, it should be noted that not all elements, molecules, or sequences can usually be fully detected. The classic omics disciplines include genomics (DNA), transcriptomics (RNA), proteomics (peptides and proteins), and metabolomics (metabolites), which together describe the flow of information from genotype to phenotype (Figure 1). For this reason, this context is often referred to as the omics cascade or as the central dogma of biology. However, RNA is mostly not very stable to environmental influences, which is why it is not usually used for the analysis of ancient artefacts. Nevertheless, sometimes ancient RNA can be detected if the right environment conditions are present [14]. In addition, it should be noted in metabolomics analyses that not only are metabolites often detected, but also other small organic compounds (<1500 Da) that are not naturally formed but synthesised by humans, the so-called xenobiotics. These can be, for example, dyes, pesticides, drugs, or cosmetics [15].

The terms genome, transcriptome, proteome, and metabolome refer to the entirety of the analytes with regard to the respective omics discipline. Based on these terminologies, other omics approaches have emerged such as metallomics (metals), isotopolomics (isotope ratios), or microbiomics (community of microorganisms. Furthermore, omics strategies are carried out without prior identification of the detected compounds, which is why they are also called non-targeted and are necessarily based on comparison with reference data. The use of non-targeted omics strategies requires high-resolution and sensitive analytical technologies to capture as many analytes as possible and to increase the information content accordingly. Since very large data sets are inevitably generated using omics approaches, bioinformatic and chemometric methods must be used for data evaluation. Bioinformatic approaches primarily include sequence analysis and are used in genomics and proteomics datasets. While chemometric methods are mainly used for pattern recognition using multivariate methods. They are suitable for the evaluation of metabolomics and metallomics data sets, but can also be used for the evaluation of genomics and proteomics data in order to visualize relationships [16].

Data evaluation should not be underestimated and, depending on the selected omics approach and the data structure, can involve a great deal of time and effort. These requirements are accompanied by an appropriate infrastructure and trained staff, which can often only be guaranteed in specially equipped laboratories. The non-targeted strategies are in contrast to the so-called targeted methods, in which analytes are known a priori and are usually also absolutely quantified. This achieves maximum comparability of the data sets but is accompanied by a loss of information. The advantage is that targeted strategies usually require less complex technological equipment. However, since no comprehensive analysis is carried out, these are not classic omics disciplines [3,13,17,18].

The analysis of the genome, proteome and metabolome is particularly suitable for organic materials such as writing surfaces made of papyrus, paper, parchment, leather, plant leaves, wood and wood bark, bamboo, silk, tapa, amate, textiles, and wax as well as inks, pigments, or dyes containing organic components. In addition to biological identity and taxonomy, further research questions may concern the production process or conservation methods, e.g., palm leaves manuscripts are protected with various plant extracts, which can be easily identified by mass spectrometric metabolomics methods [19]. In addition, such studies can be used to record similarities and differences, for example, to determine whether certain fragments or pages belong together, or to assign geographical origins [20,21,22]. In particular, the meta-information obtained with these techniques can provide indications of bacterial or fungal infestation of manuscripts, which can be helpful to stop biodeterioration processes and also to protect the people who handle the analysis material [23,24,25]. In addition, they are also suitable for obtaining information about the authors or the readers [26,27,28,29,30,31,32]. The analysis of certain stable isotopes using isotopolomics approaches can provide further information. The ratio of ^14^C and ^12^C, which can be applied to date artefacts, is of particular importance. It should be noted, however, that this method can only date the material used, not when a manuscript was actually written by an author [33,34]. Metallomics strategies including certain isotope ratios of metals are mainly suitable for analysing inorganic writing materials such as metals, stones, ceramics, clay tablets, glass, or bones [35]. They are also of great relevance for the analysis of inorganic components in pigments, dyes, and inks [18,36].

By combining the various omics levels, a comprehensive elementary and molecular profile can be created about an object and a maximum depth of information can be generated. This procedure is also called panomics or multi-omics profiling and is used, for example, in medicine or in the analysis of food (food profiling). In the context of manuscript analyses, we propose the terms “manuscript profiling” and “artefact profiling” to cover not only manuscripts but also other objects that play an important role in the context of cultural heritage such as images, sculptures and other ancient manmade objects items [3,37,38]. The analysis of ancient artefacts and manuscripts using omics strategies is still a relatively young field of research, and the terminology is evolving accordingly. Table 1 lists some of the most important terms.

In addition to the analytical methods explained in this publication, there are numerous archaeometric approaches to deciphering information about ancient manuscripts. This also includes, e.g., physical methods such as computed tomography as well as various microscopic methods, which, however, do not fall within the scope of panomics applications and are therefore not considered in this review. For the same reason, we have not gone into detail about low-resolution technologies either, as they typically capture only a fraction of the information compared to the high-resolution technological platforms used for typically omics investigations, although the boundaries can certainly not be drawn clearly.

## 2. Sampling Strategies

A challenge with all higher resolution archaeometric approaches is the sampling. Measurements directly on the object (in situ) offer an alternative, but these usually operate in a lower resolution range. In this context, a total of three approaches are being pursued as follows: non-invasive, minimally invasive, and invasive. (i) The gentlest methods are non-invasive approaches that do not require sampling or contact between the instrument and the object. However, even with such procedures it must be considered that long-term damage to the artefacts can occur; for example, if the objects are exposed to ultraviolet (UV) light for a long time, the ageing processes can be accelerated. (ii) With minimally invasive methods, sampling or contact with the measuring instrument is not macroscopically visible. In contrast, invasive approaches require either close contact with the object or sampling that is macroscopically visible. In addition, these procedures can be categorised into non-destructive and destructive methods. Non-destructive methods require that part of the object is removed but not destroyed and made available for further analysis. This is the case, for example, with fibre analyses. Using destructive approaches, on the other hand, the sample material is completely destroyed when, for example, the mass spectrometric measurements are carried out [18]. In general, the greatest information density can be generated with invasive methods precisely because they can be used not only to detect analytes on the surface, but also those elements, molecules, or sequences in the deeper layers. In addition, there is a larger quantity of analytes obtained in this way, so that the sensitivity of the analytical methods increases accordingly. However, since the greatest damage can also be caused with invasive approaches, a careful cost-benefit analysis is required to determine which extraction method is best suited. An alternative may be to use small pieces that naturally fall off old objects for analysis. In most cases, about 2–5 mm^2^, respectively a few milligrams, are already sufficient to perform the corresponding analyses [19,45,46,47,48,49].

### 2.1. Sampling Strategies for High Resolution Omics Approaches That Can Be Performed on Site

Most of the high-resolution analytical methods listed in Figure 1 typically require minimally invasive or invasive sampling. Furthermore, in most cases the sample is also destroyed or must be prepared specifically for the measurement, so that no further measurements are possible with this part. Since sampling is one of the most challenging aspects of working with ancient objects, much research has been performed in this area in recent years and alternative sampling techniques have been developed. In addition to the classic use of adhesive tapes, scalpels to scrape the surface, sponges, and swabs, a micro-aspiration technique has recently been introduced that allows molecules to be picked up from the surface with a simple vacuum [45,50,51,52,53,54]. A more destructive alternative is the simple and inexpensive polyvinyl chloride (PVC) rubber method described by Fiddyment et al. [2,55]. Promising results were also obtained using a film of ethyl vinyl acetate (EVA) in which C8 and/or C18 resins as well as cation/anion exchangers or metal chelators were fused. Before use, the film is moistened and placed on the manuscript so that the corresponding analytes are adsorbed. About 1–2 cm^2^ of the surface of the samples are covered with the film [27,56,57]. Some research groups have also had good experience with hydrophilic nanogels, which can be enriched with a variety of solvents, as well as enzymes such as trypsin (see Section 4). The area that the authors of this study covered with the gel was only 3 mm^2^ [58]. Another approach is based on the use of the fungal protein hydrophobin Vmh2, which can be obtained from the edible fungi *Pleurotus ostreatus*. The protein is placed on a cellulose loose acetate surface covering an area of 2 cm^2^. Hydrophobins are characterised by their surface-active and amphiphilic properties. However, they are currently still expensive as they are not produced on a large scale [59,60]. Further experiments were carried out with a polishing film that was applied to polystyrene rods with a diameter of a few millimeters. The film was carefully rubbed over the samples. During this process, the analytes were absorbed by the film and could then be analysed [61]. Moreover, it may already be sufficient to take up analytes from the surface of a manuscript with small volumes of solvent and then to analyse the solution obtained [62].

For the analysis of small organic molecules and metabolites by mass spectrometry (MS), the atmospheric solids analysis probe (ASAP) procedure can be used. For this approach, a glass capillary probe is rubbed over the surface of a manuscript and the adhering analytes are then introduced into a mass spectrometer via an atmospheric pressure chemical ionisation (APCI) source. The glass capillary probes can also be shipped, which is why this procedure can be carried out on site, e.g., in libraries or private collections. By using a APCI source, analytes <2000 Da can be detected [63,64]. In probe electrospray ionisation (PESI) technology, a similar approach is taken, but it can be assumed that larger molecules are still detectable since an electrospray is generated. The difference is that instead of a glass capillary, a metal needle (similar to an acupuncture needle) is used for sampling. By moistening and by applying a high voltage to the needle, an electrospray is generated, which leads to the ionisation of the analytes, and which can then be analysed using a mass spectrometer. According to the best of our knowledge, this approach has not yet been carried out on ancient objects, but on various biological samples [64,65,66]. However, it could still be a suitable alternative in the future. Further possibilities for on-site sampling are provided by special laser systems, which are discussed in Section 2.2, due to the technological requirements.

Not all the options listed here have been tested on manuscripts at this point, though some have also been tried out on paintings, for example, which is why it is currently not possible to make a conclusive assessment as to which area sizes need to be sampled. In addition, such considerations must also take into account the material used, the state of preservation of the manuscript, and the aim of the study in order to decide which sampling method is the most appropriate in each case.

Although all these methods are nearly non-invasive or minimally invasive, curators often have reservations about allowing sampling because some of these methods require the surface of the manuscript to be moistened. However, these methods also have the advantage that sampling can be carried out quickly and easily as well as on site so that the historical documents or measuring equipment do not have to be transported at great expense. In addition, immovable objects such as gravestones or inscriptions on walls can also be sampled. Although this type of sampling is normally invisible to the naked eye, slight colour changes can still occur, which should be carefully checked in advance.

### 2.2. Sampling Strategies for High Resolution Omics Approaches That Require No Sample Preparation but Must Be Performed in a Laboratory Environment

In contrast to the on-site strategies described, there are micro-invasive alternatives that require a laboratory infrastructure. The advantage of these techniques is that they enable spatial resolution and require no sample preparation. These include, in particular, ambient ion sources for coupling to mass spectrometers such as direct analysis in real time (DART), desorption electrospray ionisation (DESI) and nanospray desorption electrospray ionisation (nano-DESI). Those designs are particularly suitable for the analysis of metabolites and small organic molecules in a mass range <2000 Da since the ionisation energy is not sufficient for larger molecules [64].

For example, minimally invasive DART-MS analyses were performed on parchment to identify potential conservation treatments. In the context of old manuscripts, it became clear that it is important not to set the temperature of the DART carrier gas too high in order to avoid damage such as burn marks on the manuscripts [67]. Further, analyses using DART-MS were also carried out to differentiate between various types of paper, although the paper was not sampled directly, but rather small pieces that had previously been separated with tweezers [68]. In addition, there is a study on different inks using DART-MS. It turned out that inks change very significantly after the first application since volatile compounds evaporate before a stable state is established [69]. However, all these studies were not carried out on very old material, which is why a conclusive evaluation of this strategy in the context of ancient manuscripts is not possible at this stage. Further research in this area is needed, although DART sources are currently not very widespread.

While DART-MS designs use plasma for ionisation, the ionisation of DESI-MS or nano-DESI-MS couplings is based on an electrospray to which the surface of a manuscript is exposed. In contrast to DESI, a second capillary is used with nano-DESI to transport the analytes to the entrance of the MS. In this way, the efficiency is improved and more stable mass spectrometric signals are obtained, which is particularly helpful when analysing old artefacts since the analytes are present in comparatively low concentrations [64]. DESI-MS approaches have been applied to ancient artefacts to detect peptides in historical flint plates and pottery shards, and to analyse inks [70,71,72,73]. However, as with the DART-MS studies, the focus has so far been on more recent samples. Nevertheless, such studies can provide indications of the extent to which a transfer to historical manuscripts is possible and what damage can occur.

In secondary ion mass spectrometry (SIMS) technology, the surface of an object is bombarded with primary ions, and the actual analytes are released as secondary ions for further analysis by an MS analyser. SIMS must be performed in ultra-high vacuum, i.e., the sample must be vacuum stable and the samples must not be too large and thick, depending on the design of the equipment used. The advantage is that both organic and inorganic components can be detected and that a depth profile can be created. A further development of SIMS technology are nano-SIMS applications, which are characterised by a particularly high spatial resolution. SIMS platforms have been widely used for the study of ancient paintings [74]. In addition, other materials such as glass or bronze were also analysed [75]. Studies carried out on ancient manuscripts using SIMS do not yet exist. However, there have been numerous studies on the analysis of inks and also one on parchment, which have so far focused mainly on recent samples, but which can serve as a starting point for further developments [76,77,78,79,80,81].

In addition, the detection of inorganic elements by means of laser ablation inductively coupled plasma mass spectrometry (LA-ICP-MS) is relatively well established for the analysis of ancient artefacts. The ablation site is not visible to the naked eye. Small spots or grooves can only be seen under a microscope. An LA-ICP-MS instrument usually consists of an ablation cell in which the object is located and can be moved in x-, y-, and z- axes. Using a focused laser beam, the analytes on the surface of the sample are transferred into the gas phase, which is transported to an ICP-MS system by means of a carrier gas (Ar or He). Different laser types (solid state, e.g., Nd:Yag or gas eximer, e.g., ArF) with different wavelengths and pulse durations can be used for the ablation process, which on the one hand have an impact on the damage to the object and on the other hand on the signal intensities. Especially when analysing precious ancient artefacts, lasers with shorter wavelengths are preferable since the damage is the least (no frayed spots appear) and the ablation is efficient and fast [82,83,84]. However, the size of the artefact is limited by the ablation chamber in the currently commercially available designs. Nevertheless, there are alternative options to overcome this disadvantage. One possibility is open and moveable ablation cells or the complete omission of a cell, i.e., the laser is used under ambient conditions. In order to ensure the exchange of air for argon, diaphragm pumps or special designs of gas exchange instruments are applied in such constructions [82,85,86]. LA-ICP-MS instruments for the analysis of historical manuscripts have in particular been used thus far to require the chemical composition of iron gall inks since the metals obtained in these colours can be catalytically active and can accelerate the degradation processes of the historical documents (see Section 6.2) [36,87].

Based on the laser systems described, portable ablation systems were also developed with which sampling can be carried out directly on site, e.g., for sampling wall inscriptions or gravestones that cannot be transported to a laboratory. Consequently, such systems are more likely to be assigned to the methods from Section 2.1. The technological similarities have been mentioned in this section. The challenge with portable systems is on the one hand the size as well as the weight, and on the other hand the technical requirements such as efficient cooling of the laser unit. Diode-pumped solid-state (DPSS) lasers with a wavelength of 532 nm and a pulse rate of <1 nm, which can be operated with air cooling, are suitable for meeting these requirements. Since these lasers can only generate comparatively long wavelengths above the UV range, highly transparent materials cannot be analysed. Nevertheless, these designs can be used to generate appropriate aerosols from suitable objects, which can be collected on filters using a membrane pump and then analysed using, for example, ICP-MS instruments. However, compared to laser-induced breakdown spectroscopy (LIBS), which is quite similar in terms of sampling technique, and X-ray fluorescence (XRF) measurements, the limits of detection achieved using this approach are in the lower ranges (see Section 6.1) [88,89,90]. In the meantime, there are some successful application examples of this portable laser system which have been carried out on antique objects such as archaeological silver, antique glass beads, or various gold objects [91,92,93].

So far, sampling techniques from historical artefacts based on laser ablation methods are mainly used for the analysis of inorganic analytes. Nevertheless, there are also ways of removing organic molecules from surfaces gently and as non-destructively as possible using laser processes. Both UV and infrared (IR) lasers are suitable for this demand whereby the water content can be relevant and with which method better results can be obtained since the IR laser initially triggers a sudden evaporation of water molecules, taking the analytes with them. The analytes can be ionised directly from the resulting aerosol, e.g., by means of an electrospray, and then passed into an MS instrument. Alternatively, the ablation cloud can be collected on site with the help of membrane pumps or as condensate on slides and later analysed in a laboratory. Depending on the type of laser used, these methods are called laser ablation electrospray ionisation (LAESI) if an IR laser is used, or electrospray laser desorption ionisation (ELDI) if a UV laser is used. In the past, both methods could be applied to detect both small polar molecules and larger peptides, as well as proteins in biological materials [94,95,96]. Intact DNA molecules could also be removed from organic materials using an IR laser [97]. At the present time, it is difficult to estimate whether a transfer of such a technical strategy to old materials is possible and how high the information content is in comparison to the other methods presented since there is currently no experience in this regard. Furthermore, it is currently not known how destructive such processes are for writing materials. However, in recent years, lasers have been increasingly used to clean paintings. It was also shown that the same type of laser (UV excimer KrF laser) applied for cleaning processes can also be applied for laser-induced fluorescence (LIF) measurements. Possibly, such developments can also be suitable for omics investigation of ancient manuscripts [98].

## 3. Paleogenomics and Metagenomics Analyses

Genome analyses, which focus on examining DNA sequences, are particularly suitable for determining the biological identity (genotyping) of plant and animal writing surfaces by sequencing the extracted DNA. In addition to the taxonomic classification, this also includes the determination of sex, breed, and degree of relationship. Furthermore, the analysis of exogenous DNA sequences allows gaining meta-information about the microbiome. Not only is nuclear DNA suitable for such investigations, but also DNA from mitochondria and chloroplasts. The latter two DNA types have the advantage of being available in higher copy numbers, which can facilitate the analysis. In addition, they are in most cases only inherited from the mother’s side, which can often make data interpretation easier. The investigation of ancient DNA (aDNA) is particularly challenging since the sequences are highly degraded (<100 base pairs) depending on the environment conditions and their age. Both endogenous and exogenous nucleases are responsible for the degradation processes. There are also environmental influences such as temperature, humidity, pH value, and UV radiation. In addition, the DNA of the writing surface can already be largely destroyed during the production process. This is the case, for example, with paper, which undergoes various chemical and physical processing steps during its manufacture, which is why the DNA can no longer be detected in most cases. Conversely, certain conditions such as low temperatures and a dry environment can have a strong preservative effect [2,99,100,101,102].

First, DNA fragmentation begins with the hydrolysis of the bases, especially the purine bases, whereby the bases are cleaved from the sugar-phosphate backbone of the DNA double strand (depurination). As a result, single-strand breaks occur in the backbone of DNA sequence and the DNA double helix breaks into smaller pieces. In addition, deamination reactions take place in which cytosine is converted into uracil and 5-methylcytosine into thymine. Furthermore, guanine can be deaminated to xanthine and adenine to hypoxanthine. Due to the conversion of cytosine to uracil, thymine is incorporated instead of guanine during the generation of the complementary counterstrand when amplifying DNA by PCR prior to sequence analysis. Thus, such reactions have the consequence that in sequence analyses, erroneous information can be generated [101,103]. However, since this reaction occurs particularly frequently in the single-stranded overhangs of the fragmented aDNA pieces, the relatively high thymine concentration can also be used to check the authenticity of aDNA and distinguish recent DNA from aDNA [102,104,105]. Further, aDNA is characterised by intra-molecular and inter-molecular cross-links. These are formed by bonds of the sugars of the DNA backbone with the amino groups of DNA bases or with amino acids of proteins (Maillard reaction). In addition, thymine dimers can form. Such modifications mean that the DNA cannot be amplified by PCR and cannot be sequenced. However, they occur comparatively little, and each DNA molecule occurs in many copies. This redundancy ultimately helps in the detection of the correct sequence. Usually, the conversion of cytosine into uracil predominates [101,103].

In addition to the high degree of fragmentation and the small amount of sufficiently long DNA sequences, the extraction of aDNA is also challenging because many other DNA sequences from various organisms (i.e., from microorganisms and animal pests) are often also extracted, which are usually present in abundant concentrations [99,100]. In order to avoid additional contamination, it is advisable to carry out laboratory work in an appropriate clean room under special protective conditions [102,106,107]. Furthermore, several software tools and strategies have been developed that can help distinguish contaminations of aDNA with modern DNA [108,109,110].

### 3.1. Analytical Procedure for Genomics Analyses

The amount of samples required for DNA analysis can vary greatly and depends primarily on the material of the manuscript and its state of preservation. In addition, it remains to be considered whether, for example, the microbiome should only be examined superficially, or whether a comprehensive characterization of the writing surface or ink should be carried out. Furthermore, the method used for the detection of the DNA sequences must be taken into account. In the case of parchment manuscripts, which have so far been researched most frequently with DNA analyses, fragments with a size of approx. 5 mm^2^ were used by most working groups. A visual impression of the required size of the fragments can be found in the publication by Piñar et al. [48,111]. From historical papyrus manuscripts, it was possible to extract DNA from pieces with a size of approx. 1 cm^2^ [112]. In the case of non-destructive work, e.g., with the rubber method, an assessment is much more difficult since only superficial DNA can be obtained, which may be heavily contaminated with microbial DNA. In such cases, we recommend using the relevant literature and comparable methods and materials as a guide.

Various protocols exist for the extraction of aDNA from historical artefacts, which are used depending on the material and the scientific question. It is difficult to estimate how much DNA and in what condition the DNA is actually still present in old materials. Furthermore, the success of the extraction depends strongly on the method. In this context, there are various studies dealing in particular with the optimization of the extraction process of aDNA [51,113,114,115].

In parts, the extraction procedures are similar to those used for the extraction of modern DNA. However, there are also some special features to consider. In general, a lysis buffer is added after DNA sampling, which can be completed using the rubber method, for example (see Section 2.1). The buffer contains detergents such as cetyltrimethylammonium bromide (CTAB) or sodium dodecyl sulfate (SDS) to destroy the secondary and tertiary structure of membrane proteins; mercaptoethanol or dithiothreitol (DTT) to cleave disulfide bridges in proteins; polyvinylpyrrolidone (PVP) to bind polyphenols and polysaccharides; and if necessary, enzymes to degrade proteins. After incubation for about 24 h at temperatures between 37 and 55 °C, depending on the temperature optimum of the enzymes used, the DNA is purified, for example, with silica columns or by two-phase extraction by means of phenol and chloroform. Subsequently, the DNA is precipitated with ethanol or isopropanol and stored in a buffer of tris-(hydroxymethyl)-aminomethanhydrochlorid (TRIS-HCl) and ethylenediaminetetraacetic acid (EDTA) as well as Tween-20 for long-term storage [51,100,102,111,116].

Since, as already described, the deamination of cytosine to uracil leads to errors in sequencing, it can be helpful to treat the DNA extract with certain enzymes to reduce error rates. Uracil DNA glycosylase (UDG) is suitable for this purpose, as it removes uracil from the DNA sequence. Abasic sites remain, which are cut off with endonuclease VIII (Endo VIII). This procedure is also referred to as uracil-specific excision reagent (USER) treatment and is sold as an enzyme mixture by New England Biolabs. However, the aDNA sequence is fragmented even more by this reaction, and the higher thymine content is lost as an authentication parameter. For this reason, it is appropriate to treat one part of the DNA extract with and one part of the DNA extract without these enzymes [117]. Additional enzymatic methods have been developed to correct other aDNA modifications. An overview can be found in the reference cited [103].

In the past, DNA sequencing was commonly performed using Sanger’s chain termination method. However, this procedure is both very time consuming and expensive. With the development of next generation sequencing (NGS) instruments, ultra-high throughput parallel sequencing have become possible. Consequently, both the costs and the time required could be reduced. A whole range of different instrument types are now available, which have significant differences in read lengths, capacities, error rates, runtimes, acquisition, and maintenance costs. Currently, instruments from the manufacturer Illumina dominate the sequencing market, with about 80% of the instruments sold [118,119]. While second-generation devices first amplify the target DNA sequence using polymerase chain reaction (PCR), which can be very error-prone, third- and fourth-generation platforms do not need this step. However, relatively long DNA sequences are required for the third-generation and fourth-generation designs [118]. Since aDNA is usually highly fragmented, platforms of the second-generation are often preferred. Nevertheless, there are efforts to concatenate short DNA molecules in such a way that they become accessible for third- and fourth-generation instruments [102,120]. However, at the present time, the newer instruments are currently still used primarily in the context of cultural heritage issues in order to obtain meta-information from recent DNA sequences, especially to record the microbiome. In this context, the small MinION platform by Oxford Nanopore Technologies plays a special role (see Section 3.3) [121,122].

The second-generation instruments are based on the shotgun and sequencing by synthesis (SBS) approach, in which the genome is first fragmented enzymatically or mechanically, since only short reads can be taken into account [118]. Due to the high degree of fragmentation of aDNA, the first step can be omitted, and the so-called library preparation can take place directly after the DNA extraction. However, the manufacturers’ standard protocols for preparing libraries are often not optimal for the analysis of aDNA, since they are associated with a high loss of the template DNA sequences [99,123]. For this reason, different strategies have been developed specifically for fragmented and damaged aDNA sequences. Classically, a double-stranded library preparation is performed in which double-stranded adapters are bound to the ends of the double-stranded DNA sequences. The DNA sequences are then denatured and bound single-stranded to a carrier material by means of the adapters, and an amplification is carried out. Depending on the NGS system, a bridge or emulsion PCR is used to be able to detect sufficiently strong signals in the subsequent sequencing. During sequencing, a complementary DNA strand is synthesised and a corresponding signal in the form of a light beam or a change in pH value, e.g., is generated when the respective complementary base is incorporated [124,125]. However, the disadvantage of this procedure is that only double-stranded DNA sequences can be detected, but aDNA sequences are often present as single strands. For this reason, a method for the production of a single-stranded library was developed specifically for severely damaged DNA sequences. This approach considers both single-stranded and double-stranded DNA sequences. For this purpose, the double-stranded DNA molecules are first denatured with heat and then a single-stranded adapter is ligated to the respective ends. The adapter contains biotin, which is bound to streptavidin-coated beads. After adding an oligonucleotide that binds to the adapter, a double strand can be generated from the single strand by means of PCR. A double-stranded adapter is tied to the opposite free end of the double strand. The biotin-streptavidin bond is broken with heat and the DNA double strand is converted into two single strands, with which amplification and sequencing can be carried out analogously to double-stranded library preparation [126]. The single-stranded library approach is both more time consuming and expensive. For this reason, various modifications were made [127,128,129]. This includes in particular the so-called single-tube library preparation procedures, in which the many individual steps otherwise required for library preparation are combined in a few steps, so that the hands-on time is reduced. In addition, DNA loss when transferring the extract from a used tube to a new tube can be minimised because fewer reaction tubes are needed overall. A prerequisite for this simplification is an optimised composition of the chemicals and enzymes required for the individual steps [129,130].

The analysis of NGS data from aDNA sequences can be very challenging and time-consuming, as only short reads are obtained, which often contain errors and artefacts. For this reason, assignments are made using reference genomes from organisms that may be closely related. De novo assembly, as can be performed using NGS data of modern DNA sequences using overlapping reads, is mostly not possible due to the short read lengths. An overview of suitable software tools that can be used for the analysis of aDNA expressions was recently published by Orlando et al. [102].

As an alternative to the described non-targeted NGS methods, target enrichment sequencing approaches are also suitable, in which previously known DNA sequences are first bound to a target-specific single-stranded DNA bait, amplified, and then detected using NGS technologies. Such procedures are cheaper and can simplify the analysis especially when only a small amount of aDNA is present, but since this is a targeted approach and no omics technique, there is a risk that potentially relevant DNA sequences will not be detected, and information will be lost [131].

### 3.2. Analysis of Endogenous DNA Sequences

In the past, endogenous genomic analysis of aDNA was used specifically to study parchment manuscripts [48,55,132,133,134]. Such analyses can help, for example, to reassemble fragments of manuscripts or archives into a whole since it can be assumed that fragments originating from the same animal belong to each other. In addition, they provide information about the way of life of people in certain regions, for example, whether cow or sheep breeding was more common or whether manuscripts were produced in other places and only later reached a distant region. Examples include the study of the Dead Sea Scrolls as well as the analysis of a New Zealand founding document to which an additional blank parchment page could be added [20,135]. Further studies in this regard were carried out on a gospel book produced using various animal species (calves and sheep) and different sexes. It was particularly noticeable that four female animals and one male animal were used for the production of the parchment. Since female animals have a higher value as breeding animals, one would not initially expect this ratio. A possible explanation could be the occurrence of a cattle disease at the time of writing the manuscript, or that female animals were deliberately chosen as sacrificial offerings for the text. Conversely, it is also possible that there was an oversupply of females, as the males were mainly used as work animals. [133]. Another example is the analysis of endogenous DNA of Slavonic codices made from parchment of different animal species [48,136].

An older study from 2002 describes the decay of endogenous DNA in papyri and suggests a half-life of DNA of 19–24 years. According to this study, DNA in papyrus manuscripts is not detectable up to an age of 500–700 years. The detection was performed on chloroplast DNA, which was first amplified by PCR as well as specific primers and then analysed by electrophoresis [112]. Since new possibilities have emerged in the meantime due to the introduction of NGS strategies, it would be useful to check this assumption with the more recent developments. Further studies on plant materials such as wood, which was used both as writing material and for book bindings, and palm leaves suggest that the extraction and analysis of endogenous DNA from degenerate manuscripts should be successful and may provide information about their origin and use [51,137,138]. To the best of our knowledge, however, written artefacts have not yet been sufficiently well researched in this context [139].

### 3.3. Analysis of Exogenous DNA Sequences

In addition to the analysis of endogenous DNA from historical manuscripts, metagenomic analyses of ancient manuscripts are performed to detect the microbiome of insects, fungi, bacteria, archaea, as well as viruses, in particular to track and, if necessary, to stop biodeterioration processes. Such analyses may also be important to protect people handling the ancient materials from potentially dangerous organism. The majority of published studies in this area are based on culture-based methods and the use of various genetic fingerprinting methods such as polymerase chain reaction denaturing gradient gel electrophoresis (PCR-DGGE). Nevertheless, NGS developments have also found their way into this research field, especially since the frequently used culture-dependent methods can only detect a fraction of the total microorganisms present in environmental samples [140,141,142,143]. NGS approaches are distinguished between metabarcoding methods, also known as amplicon sequencing, and whole metagenome shotgun sequencing (WMS) strategies. In the former, only specific marker genes are sequenced by NGS strategies and matched with a database. For the taxonomic determination of bacteria and archaea, the highly conserved 16S rRNA sequence, and for fungi, the internal transcribed spacer (ITS) regions 1 and 2 as well as the 5.8S rRNA, 18S, and 28S sequences, are particularly suitable. In contrast, in WMS, the total DNA that can be extracted from an organism or sample is used for sequencing. In this way, WMS allows not only taxonomic classification, but also identification at the species or strain level. However, due to the enormous amount of data obtained in this approach, data analysis remains a challenge [144,145]. In addition to analysing the microbiome, examining exogenous DNA sequences can also be used to obtain information about the authors and readers. For example, it is possible to deduce the frequency of use of the manuscripts based on the amount of human DNA, although special care must be taken to determine whether it is modern DNA or aDNA in order not to draw false conclusions [133].

Numerous microbiomes analyses have been carried out on manuscripts made of parchment. A compilation recently published can be found in Piñar et al. [146]. One of the first studies performed with NGS on parchment manuscripts concerned the appearance of purple spots. According to Migliore et al., halophilic archaea are responsible for the purple spots that appear on some parchment manuscripts over time. It is assumed that parchment comes into contact with numerous microorganisms during the manufacturing process. These include halophilic and halotolerant microorganisms that enter into the parchment through treatment with sea salt. Under the right conditions, the archaea can reproduce and gain energy from bacteriorhodopsin and light. In the process, they also attack the collagen structures. With a decrease in salinity and an increase in humidity, they collapse, and purple stains remain. At the same time, more marine bacteria begin to grow, feeding on the remains of archaea and the attacked collagen matrix. Gradually, more and more bacteria and fungi can colonise, causing the collagen to break down so that the manuscripts begin to disintegrate [24,147]. Another research group held Actinobacteria and *Aspergillus* species responsible for the appearance of the purple spots. The different findings are not necessarily mutually exclusive since presumably various microorganisms colonise manuscripts at different times [46].

Other microorganisms detected are mainly associated with the human microbiome. These include, for example, propionibacteria as well as *Staphylococci* and *Streptococci*. It is suspected that the bacteria infected the manuscripts through handling and use, but also through kisses. Such bacteria can also be involved in the decomposition of the manuscripts [48,133,146].

In addition, viruses were also detected in parchment samples, which only became really possible with the use of NGS technologies [146]. Some of the viruses (*Siphoviridae*) could be identified as bacteriophages, the occurrence of which was associated with colonisation of the parchments by certain bacteria [48,136,146]. Other viruses such as Merkel cell polyomavirus are associated with the human “virobiota” and can therefore be traced back to contact with human skin [48,136,146].

Paper manuscripts are particularly prone to fouling by fungi, which release enzymes such as cellulases, amylases, gelatinases, proteases, and lipases that lead to degradation of the historical materials by attacking the cellulosic structures. Furthermore, fungi often form acidic metabolites that can further degrade the chemical structures of the paper through acid hydrolysis. In addition, there are stains and discolorations due to fungal infestation, as chromophoric molecules are released. The stain-causing fungi that colonise paper include, in particular, *Aspergillus* and *Penicillium* [148]. Since paper such as parchment is a hygroscopic material, it is therefore particularly important that the documents are stored in suitable conditions, avoiding temperatures above 23 °C and humidity above 65% [140,141,149]. In most published studies on paper manuscripts in recent years, culture-dependent methods or fingerprinting approaches have been used, too [141,142,148,150,151]. However, in a recently published study on a paper manuscript from the 11th century, it could be shown that the combination of culture-dependent methods and NGS methods in particular has significant advantages since the two approaches complement each other and provide complementary information [25]. A similar result was obtained from the analysis of a book, in which a higher level of biodiversity could be detected using a culture-independent NGS approach that targeted the 16S and 28S rRNA genes [152].

Microbiological examinations of papyrus manuscripts have so far hardly been carried out, even if there are numerous attempts to stop the microbiological deterioration of them by applying various substances [153]. To our knowledge, there are no studies available in connection with NGS for the detection of microorganisms. There is only one study that was recently published to identify fungi using a culture-dependent approach. The authors describe this study as the first of its kind and show how little is actually known in this context. [154].

Furthermore, some studies were conducted on wax seals, also to better understand biodeterioration processes. Bacteria as well as various fungal species were detected. NGS methods were applied in the studies so as to be able to detect nob-cultivable microorganisms as well. In addition to a second-generation NGS system (MiSeq Illumina), the MinION platform by Oxford Nanopore Technologies was used to analyse bacterial 16S rRNA and fungal ITS as well as 28S rRNA genes [155,156,157]. The Minion platform is a small and portable sequencer, the size of a small glasses case, which is based on the so-called nanopore technology. Sequencing with this design is comparatively inexpensive, and can be performed directly on site. By measuring current changes, the nucleobases can be detected, and the sequence can be read out. Comparatively, long reads can be sequenced with this technology, but the error rate is quite high, which can be reduced by increasing the coverage or, in the case of double-stranded DNA molecules, by using a duplex approach [118]. Currently, this technology is more suitable for the sequencing of modern and exogenous non-fragmented sequences and is therefore well suited for microbiome analysis, but not for aDNA [121,122].

## 4. Paleoproteomics and Metaproteomics Analyses

The first investigations of amino acids in ancient fossils date back about 70 years. However, the big breakthrough came only with the use of soft-ionisation mass spectrometer instruments at the beginning of 2000 by Ostrom et al. [158]. Until then, peptides or proteins had to be analysed using gel-based techniques and protein sequences determined using Edman sequencing, which is time-consuming, expensive, and limited to a length of around 20 amino acids. Compared to the investigations that are carried out on aDNA, paleoproteomics analyses are still a comparatively young field of research [159].

The results obtained from proteomics studies show some overlap with those from genomic analysis (Figure 1). Thus, proteomics approaches can also be used to investigate the biological identity of manuscripts. In contrast to DNA analysis, the taxonomic resolution is not as high, e.g., it is not possible to determine gender or the degree of relationship. However, proteomics analysis can be applied to identify different types of tissue, e.g., if egg yolk or egg white was used. In addition, proteomics analyses are suitable for the detection of proteinaceous residues, e.g., from paint binders, glues, and certain surface treatments [13,159,160]. The major advantage of proteomics compared to genomic analysis, especially for historical artefacts, is that ancient peptide sequences are more stable and may still yield results when aDNA sequences are too fragmented to analyse. The stability of proteins depends on various parameters. On the one hand, this includes environmental factors because, similar to DNA molecules, high temperatures, extreme pH values, moisture, or enzymes can lead to the degradation of the protein sequences. On the other hand, the primary and thus the secondary, tertiary, and possibly quaternary structure also have a major influence on stability, which can lead to some proteins being broken down more quickly than others. Compared to DNA molecules, proteins are also present in larger quantities. However, unlike genomics techniques, it is not possible to carry out an amplification of the analytes. The qualitative and quantitative occurrence of proteins depends on various endogenous and exogenous factors. This means that compared to DNA sequences, greater diversity must be expected, which can have both advantages and disadvantages: on the one hand, this diversity makes data analysis more difficult, and on the other hand, in the best case scenario, additional information about an object is obtained [2,13,159,160]. The performance of proteomics is faster and cheaper compared to DNA analysis. However, since genomics and proteomics studies often provide complementary information, both techniques are used at best [2,13,159]. A procedure that is already being carried out by some working groups [55,133,152].

During the ageing of proteins, fragmentation occurs through hydrolysis of the backbone of the protein sequences, in which the peptide bonds are cleaved. Smaller peptides or free amino acids are also obtained as reaction products [161]. Further, ageing reactions can affect the side chains of the amino acids. The focus here is on the non-enzymatic deamidation reactions of glutamine to glutamic acid and asparagine to aspartic acid, which can be used to distinguish between ancient and modern proteins and sometimes even as a tool for estimating ageing (“molecular clock”). However, it must be noted that environmental factors and the protein structure can have a strong influence on such reactions. In addition, deamidation reactions can also occur in vivo, which is why the results are sometimes not very meaningful [161,162,163,164,165]. Nevertheless, it has recently been proposed to use the deamidation grade of glutamine for the calculation of a Parchment Glutamine Index (PQI). However, the focus is not on the time-dependent deamidation, but on the production process, in particular the liming with Ca(OH)_2_, the animal type used, and the thickness, since these factors also have an influence on the deamidation grade [166].

Except for glycines, all proteinogenic amino acids have at least one chiral carbon atom and can occur in the L- or D- conformation. In most cases, amino acids occur in biological systems as L-isomers. The conversion of L-amino acids to the corresponding D-enantiomers is called amino acid racemisation (AAR) and can also be used as a marker of ageing. However, these reactions are also influenced by many exogenous factors and the protein structures, which must be taken into account for a reliable assessment [161]. In addition to the reactions described, numerous other degradation and conversion processes can occur during the ageing of proteins. These include chemical processes such as Maillard reactions, dehydration, decarboxylation, lactamisation, aldol cleaveage, oxidation, phosphorylation, and dephosphorylation as well as hydroxylation and dehydroxylation. Additionally, there are enzymatic degradation reactions by the microbiome. Such reactions make the data more complicated, but can also serve as important authenticity markers for distinguishing between modern and ancient sequences [161].

### 4.1. Analytical Procedure for Proteomics Analyses

**Figure 2:**
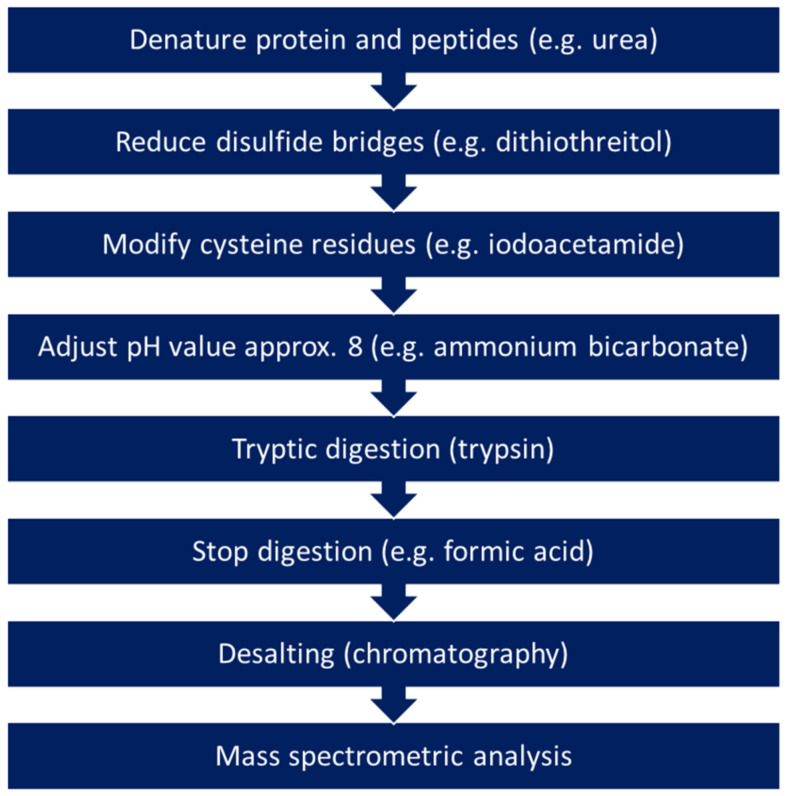
Typical sample preparation steps for performing bottom-up analysis.

Proteins or peptides can be extracted from ancient manuscripts either directly from smaller fragments or using the methods described in Section 2 such as the rubber method, the use of EVA films, hydrophobin, or gels. Researchers that prefer destructive sampling for proteomics analyses also use small fragments with a size of approx. 5 mm^2^ or about 5 mg, similar to DNA analyses [134,167]. The proteins and peptides can then be dissolved with different buffers. For further analysis, two different approaches are followed, referred to as bottom-up and top-down strategies. As in many other research areas, proteomics analyses of ancient manuscripts are usually carried out using the bottom-up approach, in which the proteins and peptides are first enzymatically digested (Figure 2). The enzymatic digestion is usually performed with the enzyme trypsin. Trypsin is an endopeptidase and cleaves peptide bonds behind the amino acids lysine and arginine at the C-terminus. The smaller peptides obtained can then be analysed using mass spectrometers and assigned to the corresponding proteins with the help of matching to databases. The disadvantage of this approach is that complete sequence coverage can often not be achieved and that fragments with post-translational modifications (PTMs), protein truncations, as well as alternative splicing events cannot be identified [13,147,168]. In the top-down approach, intact proteins are analysed so that proteoforms can also be characterised.

However, there are some physical and technological limitations with this approach. On the one hand, these result from the fact that the extraction and purification of intact proteins is significantly more difficult because there are in many cases limits caused by their solubility, which is why some of the sampling methods described in Section 2 also suggest in situ digestion with trypsin. On the other hand, there are limitations set by the mass spectrometric detectors since the sensitivity of most detectors is inversely proportional to the mass of the proteins. However, some efforts currently exist to overcome this disadvantage [169]. The extent to which there are still really intact proteins in ancient manuscripts is questionable and, as already mentioned above, depends on the material used and on the exogenous factors during storage. Nevertheless, such an approach could certainly provide information about the composition of very stable molecules such as collagen. Furthermore, information about the degree of degradation of the ancient materials could be obtained [13,170]. In addition to these two classic approaches, there is also the middle-down proteomics approach, with which the low sequence coverage of the bottom-up approach can be improved, and simultaneously it is ensured that the limitations of the top-down approach are reduced. In the middle-down approach, the enzymatic process is carried out with proteases, which do not work quite as efficiently as trypsin, so that larger peptides are obtained. This strategy is relatively new and to our knowledge, has not been used to research ancient manuscripts [13,171]. 

Mass spectrometric analysis of proteomics experiments is performed in most cases with matrix-assisted laser desorption/ionisation time-of-flight (MALDI-TOF), liquid chromatography electrospray ionisation (LC-ESI)-Orbitrap, or liquid chromatography electrospray ionisation-quadrupole time-of-flight (LC-ESI-QTOF) instruments. Both MALDI and ESI are soft ionisation techniques that largely avoid initial fragmentation of the analytes. MALDI and ESI sources are complementary to each other. In the best case, both techniques are used to obtain as much information as possible about a sample, especially since both technologies can cause ion suppression effects, which can lead to analytes not being detected [13,168]. To reduce ion suppression effects in MALDI and to achieve the highest possible ion yield, so-called MALDI-2 methods have been developed in recent years. In this procedure, normal desorption and ionisation of the matrix as well as analytes first take place using a UV laser. Immediately afterwards, when the MALDI plume has formed, post-ionisation is carried out with a second laser in order to achieve a charge transfer from post-ionised matrix molecules to neutral analyte molecules [172,173]. This technique should be particularly useful for examining extracts from ancient artefacts in which only low concentrations of the analytes are present, either because a large part has already been degraded or because, for example, the sampling was non-invasive and only a few analyte molecules are present. However, although this strategy was developed a few years ago, it has only recently become commercially available and, to our knowledge, no studies of ancient artefacts or manuscripts have yet been published.

To minimise ion suppression in ESI-based instruments, the analytes are usually pre-separated using capillary electrophoresis- (CE) or LC-systems. NanoLC systems are particularly suitable for this purpose. In addition to improving analyte coverage due to the increase in sensitivity, these systems have the advantage that the amounts of solvent required can be reduced, which has a positive effect on costs and sustainability [13,174]. The coupling of a nanoLC-ESI system with an orbitrap analyser enables platforms with a particularly high resolution and sensitivity. Orbitrap analysers, currently only available for sale by Thermo Fisher, are often used as hybrid instruments by implementing a quadrupole or an ion trap. The newer designs, known as Orbitrap tribrid mass spectrometer, have three mass analysers. These are a quadrupole, an ion trap, and the orbitrap. Together, they enable various multiple analyte fragmentation options, which simplifies structure elucidation by recording MS/MS or MS^n^ spectra [175]. Such designs are particularly suitable for bottom-up approaches in which a peptide fragment fingerprinting (PFF) analysis is performed. In this way, sequence information can be obtained and, if necessary, de novo sequencing can also be carried out. Different strategies are pursued for the recording of MS/MS spectra, such as data-dependent analysis (DDA also known as information dependent acquisition (IDA)) or data independent analysis (DIA). Typically, in non-targeted measurements, DDA experiments are performed, and MS/MS are recorded from the most abundant ions. In order to ensure the best possible coverage of the various precursor ions by means of MS/MS spectra, decision or exclusion algorithms are often used. However, it may still be the case that only the most intense signals are captured, and important smaller signals are not fragmented. This risk can be reduced by means of DIA experiments and the application of strategies such as elevated-energy mass spectrometry (MS^E^, supported by Waters, Milford, CT, USA), all-ion fragmentation mode (AIF, from Thermo Fischer Scientific, Waltham, MA, USA and Agilent Technologies, Santa Clara, CA, USA), parallel accumulation serial fragmentation (PASEF, supported by Bruker, Billerica, MA, USA) or sequential window acquisition of all theoretical mass spectra (SWATH, introduced by Sciex, Framingham, MA, USA). However, the evaluation of the data is more complex [44].

In contrast, in peptide mass fingerprinting (PMF) experiments, full scan spectra are recorded, and the peak lists obtained are compared to appropriate databases to carry out identification. This procedure is relatively simple and fast, but has the disadvantage that the corresponding databases must be available. For the latter approach, MALDI-TOF designs are often used, too [13,159,160].

TOF analysers can be coupled using both ESI and MALDI sources. In the meantime, dual platforms are also available that can be operated with both sources and can therefore be used more flexibly. When coupling a TOF analyser with an ESI interface, an LC or nanoLC unit for separating the sample extracts and a quadruple are usually also installed in order to be able to carry out fragmentation experiments. One of the most disruptive developments in recent years regarding the construction of such LC-ESI-QTOF-MS platforms is the integration of ion mobility (IM) cells, with which the ions can be separated according to their size, shape, and charge using electric fields and a drift gas. In this way, isomers and isobaric molecules can also be distinguished from each other. In addition, the collision cross section (CCS) value is received as a further identification parameter and the MS/MS spectra rate can increased which can be helpful when performing PFF experiments [44,64]. Carrying out proteomics studies, the use of IM cells is suitable for both top-down and bottom-up approaches to improve sensitivity and the number of detectable features. Although LC-ESI-QTOF-MS instrumentation currently plays a minor role in proteomics analysis because the mass resolution is not comparable to orbitrap designs, the implementation of IM cells offers a high added value that makes these designs interesting for proteomics experiments [176]. There is also the possibility to upgrade Orbitrap analysers with field asymmetric ion mobility spectrometry (FAIMS) cells, but these IM cells have a different physical principle than the usual IM cells of TOF analysers, which is why, for example, no CCS values can be generated [44,64]. Other suitable mass analysers are fourier-transform ion cyclotron resonance (FT-ICR) designs and, under certain circumstances, triple quadrupole (QqQ) or QTrap couplings. The latter two are used when analytes that are already known are to be detected and, in most cases, also to be quantified, since they are particularly sensitive. However, this is a targeted approach and not a non-targeted omics procedure. FT-ICR analysers have the best mass resolution and mass accuracy, but scan rates are comparatively slow, making coupling with LC techniques cumbersome. They are also relatively expensive to purchase and maintain, which is why they are not very widespread. An overview of the various advantages and disadvantages of the various analysers can be found in the references given [175,177,178].

The introduction of mass spectrometric methods for the analysis of proteins and proteins has brought many advantages, but it also has some disadvantages. This includes the fact that many signals cannot be correctly identified because the signal-to-noise ratio is too low, databases are incomplete or unexpected PTMs are present. In addition, fragments of sequences can be detected, but not the complete sequence itself. All these factors together mean that only a fraction of the information can be obtained. Especially when analysing ancient proteins or peptides, it can be assumed that these disadvantages have a particularly negative effect. To overcome these drawbacks, efforts are currently being made to make the method described in Section 3.3 for sequencing DNA and RNA using nanopores accessible for protein sequence analyses. At the moment, there are still a few challenges in development that need to be solved. Nevertheless, such a strategy could take proteomics investigations to a new level in the future [179].

### 4.2. Analysis of Endogenous Proteomics Sequences

Endogenous proteomics analyses are particularly suitable for the rapid taxonomic determination of the animal species used in parchment manuscripts. The first studies in this regard were carried out on a pocket Bible, also known as the “Marco Polo Bible”, from the 13th century, as well as on a Qur’an folio from the 9th century. In both studies, sampling was performed destructively by removing small pieces of parchment [167,180]. Just a few years earlier, in 2010, Buckley and Collins coined the term “zooarchaeology by mass spectrometry” (ZooMS) and described the identification of animal species from proteinaceous material using characteristic peptide sequences of collagen I by means of an PMF approach [181,182]. In 2015, this method was further developed and minimally invasive sampling with the PVC rubber mentioned in Section 2.1 was suggested. In this method, collagen molecules or fragments are detached from the surface by careful erasing and bound to the rubber residues using the triboelectric effect. Subsequently, analytes can in turn be extracted from the erase residues, digested using trypsin and analysed by a MALDI-TOF instrument. The authors of this study refer to this procedure as electrostatic zooarchaeology by mass spectrometry (eZooMS) [55]. Recently, this approach has been extended by an automated data evaluation procedure [183]. Since the first application, several studies have been carried out on manuscripts from parchment using the eZooMS approach. In the majority of publications, genomics- and proteomics-based methods were used to take advantage of both omics strategies. The results obtained allow conclusions to be drawn about the animal species used and thus in turn interpretations of geographical origin, production processes, and livestock [55,133,134,184,185,186,187,188]. In addition, certain animal skins were probably used deliberately, either because of price differences or because of their practical utility. For example, legal deeds from thirteenth to twentieth century in the United Kingdom were probably mostly made of sheepskin because fraudulent changes can be more easily traced on it [189]. Furthermore, proteomics analyses are suitable for the investigation of illuminated manuscripts and inks since these were often produced with proteinaceous binders and glues, e.g., from the egg yolk and/or egg white of various birds, as well as from gelatin, collagen, or milk of various animals and certain plants, e.g., gum arabic [12,190].

The examples listed show that proteomics analyses are particularly suitable for the analysis of protein-rich materials such as parchment or leather. However, it was recently possible to extract proteins from Tibetan paper fragments and thus draw conclusions about the manufacturing process. Proteins from *Stellera chamaejasme*, which often served as a plant basis for Tibetan paper, could be detected, as well as proteins from milk and wheat, which probably served as binders and fillers [191]. However, it must not be forgotten that paper is often very highly processed, and it can therefore be difficult to identify peptide sequences still present in the paper, which is why proteomic analyses are not always promising depending on the type of paper production [2].

### 4.3. Analysis of Exogenous Proteomics Sequences

Analysis of the exogenous proteome of writing artefacts can be used to characterise the microbiota, as in genomics-based studies. In a direct comparison between genomics- and proteomics-based studies, almost the same results were obtained. However, a better characterisation of the microorganism was possible with the NGS technologies, which is partly due to the currently available databases [152,192]. To date, comparatively few proteomics studies were carried out to identify microorganisms for the investigation of ancient artefacts. In other areas such as food analysis and medical issues, proteome-based approaches have already established themselves as a standard method for detecting microorganisms [193].

In addition to examining bacteria that settle on ancient manuscripts or that normally occur on the human skin flora but are relatively unspecific, bacterial residues were also identified in some cases, which allow some conclusions to be drawn about the authors of the manuscripts. This includes, for example, a study conducted on a typewritten letter by George Orwell. A bottom-up analysis was used to identify peptide markers that confirmed that Orwell was a carrier of *Mycobacterium tuberculosis* [30]. On the basis of an original manuscript by the Russian satirist Mikhail Bulgakov, it could also be proven that the author died of nephrotic syndrome. This study was preceded by other investigations in which morphine was detected. However, it was not initially clear whether the morphine entered the manuscript through consumption by the author or by other people. With the detection of protein markers specific to kidney disease, the authors of the two studies concluded that morphine was used by Bulgakov himself to alleviate his suffering [27,28,56]. Further proteomic investigations were carried out on the death registers from Milano of 1630. In addition to peptides that could be used to identify *Yersinia pestis* pathogens, anthrax proteins were also unexpectedly detected. In addition, numerous other peptide markers could be identified that originate from humans, mice, and rats and indicate vegetable protein residues from potato, corn, rice, carrot, and chickpeas, which presumably came from the authors’ meals [26]. Protein residues from foodstuffs such as honey, eggs, cereals, milk, and legumes have been detected on parchment manuscripts used as birth girdles. Presumably, these foods were used for treatment during pregnancy and childbirth. In addition, the scientists also found numerous human proteins, probably from vaginal secretions, among other things, which indicates a practical use of the parchment rolls [188].

## 5. Paleometabolomics and Metametabolomics Analyses

As the end product of genetic and enzymatic processes, metabolites are closest to the phenotype and at the last stage of the omics cascade. In addition to endogenous factors, the presence and absence of metabolites as well as their various concentrations within an organism are particularly influenced by exogenous factors. Compared to the proteome, the metabolome shows stronger changes to exogenous factors because metabolites often serve as inhibitors and activators for enzymes and changes in gene expression or enzyme transcription are slower, so that modifications of the proteome do not always correlate with changes in the phenotype. In animals, relevant exogenous factors can be, for example, different food sources, while in plants, the climate and the availability of nutrients play a role. In addition, metabolome analyses are also suitable for detecting xenobiotics, i.e., analytes that are foreign to the organism such as drugs, cosmetics, or organic dyes, as already mentioned in Section 1. Metabolomics analyses are relatively widely used in the analysis of foods to distinguish geographical origins, different varieties or cultivation, storage and processing conditions. In addition, they are often applied for medical issues, e.g., to detect certain diseases and to understand the course of the disease [3,17,44,64]. However, the use of truly non-targeted metabolomics approaches for the analysis of ancient manuscripts is currently the least common. This circumstance is probably also related to the fact that metabolites are often not very stable to exogenous factors and can be broken down and transformed quickly, which can make interpretation of the data more difficult.

Nevertheless, some studies have recently been published in this context, as is made clear in Section 5.1 and Section 5.2. However, so far, the part of the studies of small organic molecules in which single selected organic molecules were in the foreground (targeted studies) predominates.

### 5.1. Analytical Procedure for Metabolomics Analyses

Metabolites can be destructively obtained directly from small pieces of the manuscript, but also with less destructive sampling strategies, e.g., EVA films or ASAP and PESI approaches, as well as with special ionisation techniques such as DART and DESI sources or with the help of laser systems (see Section 2). The amount of sample required for metabolomics analysis can vary greatly when working destructively with fragments. While a few µg of sample material are sufficient for pyrolysis–gas chromatography (Py-GC-MS) analyses, 5–50 mg of the samples are required in most cases for gas chromatography–mass spectrometry (GC-MS), liquid chromatography–mass spectrometry (LC-MS), or nuclear magnetic resonance (NMR) spectroscopy analyses [19,190,191,194,195,196]. It may be possible to further reduce the amount of sample required by using new technologies such as nanoLC-MS couplings [64]. An alternative could also be to perform different analyses from the same extraction approach. Such a procedure has been performed, for example, at a mural for the analysis of lipids and proteins by exploiting the different solubilities of the analytes [197].

Solvents such as water, acetonitrile, methanol, isopropanol, or chloroform are suitable for extracting metabolites, for example, from the EVA film or from small pieces. The selection of the appropriate solvent depends on the physical and chemical properties that are the focus of the analysis and the device platforms applied. In order to bring as many metabolites as possible into solution, it can also be helpful to use methods for cell disruption, e.g., ball mills or ultrasonic waves [21].

Usually, MS and NMR spectroscopy platforms are used for non-targeted studies of metabolites (Table 2). For non-targeted investigations of metabolites using MS, the high-resolution mass analysers already mentioned in Section 4 such as QTOF, Orbitrap, or FT-ICR are generally applied. These are often also coupled with LC or LCxLC, nanoLC, or CE units via ESI sources. Alternatively, MALDI-TOF instruments can be used to analyse metabolites, or surface-assisted laser desorption/ionisation (SALDI)-TOF experiments can be performed [3,44]. SALDI is a modification of MALDI. The organic matrices typically used in MALDI experiments often lead to interferences in low mass ranges, which makes it difficult to detect the actual analyte molecules. With SALDI-based approaches, inorganic matrices are usually used, with which these interferences can be avoided, which is why SALDI is more suitable for the analysis of metabolites [151]. In addition, the chromatographic separation of metabolites can be carried out using GC or GCxGC couplings if the analytes are correspondingly volatile or have been chemically derivatised beforehand. GC-MS approaches have the advantage that they allow headspace analysis to investigate volatile compounds without having to destroy the samples [198]. Another option for rapid sample preparation is to use Py-GC-MS platforms. With these instruments, small sample quantities of a few µg are heated to decomposition and then the thermal degradation products are characterised using GC-MS [194,195].

The GC units are usually coupled to a mass spectrometer via electron ionisation (EI) or chemical ionisation (CI) sources. However, a higher number of analytes can usually be detected with LC-MS couplings compared to GC-MS-based platforms. To our knowledge, no comparative studies have been carried out on ancient artefacts in this context, but on other matrices so that the results can be transferred accordingly [44,64].

Mass spectrometers are very sensitive and allow the detection of analytes with a wide variety of chemical and physical properties, as long as they can be ionised. However, mass spectrometric measurements that are carried out at longer time intervals are not very reproducible, which can make it difficult to compare the acquired data directly. There are various strategies how to deal with the low reproducibility, especially in the case of high-resolution non-targeted measurements [44]. Alternatively, NMR spectroscopy instruments are also suitable for use in metabolomics studies, which can deliver repeatable measurement results over long periods of time. NMR spectroscopy devices, however, are less sensitive and usually only small numbers of different analytes can be recorded with them, which can be crucial when studying old manuscripts if only a small amount of sample is available anyway. Nevertheless, it can be helpful to use MS and NMR spectroscopy platforms in combination since they can be used to detect different metabolite classes [199,200,201].

In addition to the relatively widespread high-resolution NMR spectroscopy instruments with field strengths of several hundred MHz, small and compact tabletop low-resolution NMR spectroscopy devices equipped with permanent magnets are also used in manuscript research. These have the advantage that they are mobile and only require a power connection with no cooling with liquid nitrogen or helium since no superconducting magnets are used [200,202]. In addition, special designs such as the NMR-MOUSE instrument enable non-destructive surface analyses and the creation of depth profiles of objects of any size as long as they are hydrogen-containing materials. However, the information content obtained with these smaller instruments is significantly lower since no non-targeted analyses are carried out to detect individual metabolites; instead, structural and moisture analyses are the priority to obtain information about the composition, condition, and age of manuscripts. Similar information can also be obtained by solid-state NMR spectroscopy (^13^C CP MAS (Cross Polarisation/Magic Angle Spinning) NMR spectroscopy) [203,204].

Metabolomics analysis data are usually evaluated using multivariate methods to extract differences between various sample groups. However, it is often quite challenging to identify the most relevant metabolites. In the case of LC-MS data, this is completed by acquiring additional MS/MS spectra, the interpretation of which requires some experience. The CCS values mentioned in Section 4.1, which are recorded using an additional IM cell, can significantly simplify this step. GC-MS data are typically acquired at 70 eV with an EI source. Since these are standard parameters, which, unlike LC-MS applications, also deliver very reproducible results, identification using database comparisons is well established. In the case of high-resolution NMR spectroscopy data, the substances are assigned either on the basis of the chemical shift, the integration of the signals and the scalar coupling, or with the aid of reference substances [178].

**Table 2:** Advantages and disadvantages of the different technology platforms suitable for metabolite detection [199,205,206,207].

Technology	Metabolite Coverage	Advantages	Disadvantages
GC-MS	- mainly polar metabolites, e.g., sugars, amino acids, organic acids as well as fatty acids after hydrolysis also from non-polar lipids - depending on the sample, several thousand metabolites can be detected	- depending on the analysis material, up to several thousand metabolites can be detected - very sensitive (approx. nmol/L-µmol/L) - relatively robust and cheap - good reproducibility	- analytes must be easily ionisable - thermally labile analytes cannot be measured - the injected sample volume cannot be measured again - the analytes often have to be derivatised
LC-MS	- polar metabolites e.g., sugars, amino acids, organic acids or fatty acids and lipids e.g., such as glycerols, phospholipids as well as sterols - depending on the sample, several thousand metabolites can be detected	- depending on the analysis material, up to several thousand metabolites can be detected - very sensitive (approx. pmol/L-nmol/L-nmol/L) - numerous ionisation sources are available to detect analytes with a wide variety of chemical and physical properties - it is possible to carry out very fast direct infusion experiments	- analytes must be easily ionisable - the injected sample volume cannot be measured again - relatively expensive to purchase and maintain - low reproducibility - limited robustness
NMR spectroscopy	- mainly polar metabolites e.g., sugars, amino acids, organic acids - depending on sample, only hundreds of metabolites can be detected	- poorly ionisable metabolites can also be detected - the sample extract is still available after the measurement and can be measured again - very reproducible results	- the detectable number of metabolites is significantly lower compared to GC-MS or LC-MS platforms - less sensitive compared to MS instruments (approx. µmol/L-nmol/L - very expensive to purchase and maintain depending on the field strength

### 5.2. Analysis of Endogenous Metabolites

So far, metabolomics studies have been used in particular to understand the degradation and ageing processes of writing surfaces. In most of these studies, not only were antique samples analysed, but also artificially aged samples that were previously exposed to higher temperatures or humidity as well as treated with acids and alkalis in order to be able to use corresponding reference standards for comparative analyses. In addition to CE-MS and NMR spectroscopy instruments, Py-GC-MS platforms were used particularly frequently in order to be able to detect specific degradation metabolites. Such studies have been carried out for all relevant organic writing surfaces such as paper [196,198,208,209,210,211,212], papyrus [213], parchment, and leather [214,215]

In addition, metabolomics studies can be applied to gain information about the materials used for the production of the writings surfaces, e.g., what plant materials were used [191,194,195,216] or whether animal ingredients were added to a predominantly cellulose-based paper. The latter can be demonstrated, for example, by analysing the amino acid profile [196].

Furthermore, metabolomics studies have been applied to the investigation of preservation methods of manuscripts. Thus, it was possible to detect the surface treatment of parchments with castor oil and glycerol by means of a DART-MS based approach using multivariate methods for data evaluation [67]. Additionally, for the study of conservation methods, but on Indian palm leaf manuscripts, a GC-MS approach was used to identify diverse plant oils acting as insecticides and fungicides [19].

Moreover, metabolomics studies have been used to study inks, e.g., to identify the natural dyestuffs or binders [12,217]. Further investigations in this context were carried out on medieval illuminated scrolls of parchment in order to elucidate the paint recipes [190], to characterise the production of orchil, a purple dye that can be obtained from various lichens [218] and on the writings of the Italian physicist Alessandro Volta. In the latter study, around 1800 metabolites were recorded using a GC-MS platform, which indicate, among other things, that the ink was produced from *Rubia tinctorum* [219].

By carrying out metabolomics analyses of different sample groups, it is also possible to determine the geographical origin of materials or to prove whether artefacts have certain similarities and differences. We were able to show this recently with wood samples, for example [21,22]. However, it can be assumed that this approach can also be transferred to other organic plant materials such as palm leaf manuscripts or papyrus manuscripts and that classification studies of ancient documents should also be possible if corresponding reference samples are available. An example of this is a study performed on ancient Tibetan paper to characterise the plant material used. The analysis was based on a comparison of an ancient paper manuscript with a modern reference sample of handmade paper [191].

In addition, metabolomics approaches can also be used for questions about authentication, as was illustrated, for example, by the works of the Scottish poet Robert Burns. The focus of this study was the examination of inks and paper in order to distinguish the author’s original works from fakes [62].

### 5.3. Analysis of Exogenous Metabolites

Like genomics and proteomics approaches, metabolomics studies of exogenous metabolites are also suitable for detecting the infestation of written artefacts with microorganisms. For example, the volatile metabolome of moulds growing on parchments was analysed using headspace GC-MS analysis to determine whether active mould growth could be detected in this way. The results should help conservators to take appropriate measures or not in order to avoid unnecessarily burdening the sensitive documents with disinfection measures [220]. Furthermore, the foxing of paper samples was analysed using a SALDI-TOF-MS approach to obtain information about the microbial mechanisms involved in foxing formation and to identify the substances that lead to staining of the paper [151]. Another study, which was also carried out using SALDI-TOF-MS, focused on bee wax seals. Some of the detected metabolites could be associated with microbiological degradation processes, too [156].

In addition, information about the authors or the readers of a written artefact can also be obtained by means of metabolomics studies. For example, a recent analysis of a booklet, a certificate for a thermometer, an article, and other personal objects belonging to the American writer Jack London, could not prove the consumption of opium, morphine and heroin. This result contradicts the relatively widespread assumption that Jack London consumed these substances regularly. However, the authors of this study were instead able to identify twelve other drugs believed to have been used by Jack London [221].

## 6. Metallomics and Isotopolomics Analyses

Metallomics analyses are particularly useful for examining inorganic materials such as metals, stones, ceramics, clay tablets, glass or bones. They are also of importance when analysing inks, as these often contain inorganic components [18,36,87]. In addition, metallomics analyses also provide important information about the toxicity of ancient objects and thus about how to handle them safely [35,36].

### 6.1. Analytical Procedure for Metallomics and Isotopolomics Analyses

Various destructive, non-destructive, and micro-destructive analytical methods are suitable for the analysis of metals from written artefacts. The non-destructive methods include XRF and PIXE, while LIBS is a micro-destructive method [18]. In addition, ICP-MS, in particular LA-ICP-MS (see Section 2.2) instruments, can also be used for metallomics approaches, which are the most sensitive. Usually, samples that are to be measured using ICP-MS are digested with acids (e.g., nitric acid) and oxidizing substances (e.g., hydrogen peroxide) to remove any organic residues before analysis. However, this step requires comparatively large sample volumes, which is why laser ablation methods have been developed. These are directly coupled with ICP-MS instruments, which means that wet-chemical digestion can be omitted [44,82].

The application of XRF instruments for the analysis of manuscripts is one of the most widely used research methods, and its usefulness has now been documented in numerous publications [18]. Using XRF instruments, electrons are excited from the inner atomic shells and ejected so that electrons fall back from the outer shells to the inner shells. During this process, characteristic fluorescent X-rays are released, which can be used to draw conclusions about the respective element. When working with X-rays, it is important to remember that the rays can cause long-term damage to the objects to be examined. Since the devices can often be easily transported, they can be used directly on site [222].

Alternatively, elemental analysis can also be performed using ion beams such as those generated by proton-induced X-ray emission (PIXE) instruments. In this technique, protons or alpha particles are directed at the ancient object, which also cause electrons to be ejected off the element’s inner shells, which in turn leads to the release of characteristic X-rays. For PIXE analyses, the samples must not be too large since they are usually placed in a vacuum chamber. However, there are now also designs that do not require a vacuum, but the lateral resolution is then lower and the detection limits increase [223]. Unlike the XRF devices, PIXE instruments cannot be transported. XRF platforms have reduced sensitivity compared to PIXE instruments when analysing light elements, which is why XRF devices are more suitable for detecting elements with higher masses [222].

In LIBS analyses, parts of the sample are ablated with a laser and transferred to a plasma at temperatures of several 1000 K. The excited atoms and ions emit characteristic emission spectra when returning to a lower-energy state, from which the composition of the sample can be deduced. LIBS instruments are also transportable [222].

In a comparative study on egg tempera pigments, oil based pigments, decorated glazed ceramics, and coins, it was shown that all three platforms (XRF, PIXE, and LIBS) have different advantages and disadvantages, which is why the authors recommend a combined analysis or to base their choice on the sample matrix, the most appropriate technology [222].

While the detection limit for XRF, LIBS, and PIXE is in the ppm range, LA-ICP-MS instruments can measure down to the ppb range. However, it must be noted that it depends heavily on the devices used and the different matrices. In addition, the instruments differ in the analytical coverage with regard to the detectable elements and in the resolving power, which is relevant, for example, if not only the individual elements are to be distinguished from one another, but also their isotope ratios. Further differences result from the lateral resolution and the penetration depth [224]. Accurate quantitation is a challenge with all of the techniques listed. In general, certified reference materials are used that have a matrix similar to that of the objects to be examined. However, such reference materials for ancient manuscripts are not actually available, so in most cases only qualitative analyses are performed [54,225,226].

Isotopolomics data are usually evaluated using multivariate analysis methods, as is the case with metabolomics analyses. However, it is not necessary to identify the variables here, which is why the data analysis can be carried out much more easily and quickly, especially since the number of detected elements is also kept within a manageable range [3].

### 6.2. Analysis of Endogenous Metals and Isotope Ratios

Metallomics analyses are part of numerous publications that have been carried out on written artefacts, as already mentioned, the analysis of the inks and illuminated manuscripts is in the foreground. More detailed information on this can be found in the recently published review by Burgio [18]. The questions of ink analyses relate in particular to their composition in order to gain information about their manufacture, but also to prevent ink corrosion, as occurs with iron gall inks, and which can lead to damage to the historical documents due to acid catalysis and/or redox reactions [36,87,227]. However, Cappa et al. recently pointed out that metal analysis should not be used alone to discriminate inks, as only the elemental compositional information is not sufficient to provide an adequate characterisation. Therefore, the authors recommend combining inorganic analyses with other methods that also record organic compounds [18,228].

Furthermore, metallomics approaches can also be used to date inks, to prove their authenticity, or to determine the geographical origin [18]. For the latter, the isotope ratios of lead, strontium, and iron have proven to be particularly suitable [229,230].

In addition, information about the writing surfaces themselves can also be obtained. For example, a high proportion of calcium in parchments indicates certain production methods and can provide information about the microbial flora [48,136]. Moreover, it is possible to distinguish newer and older documents from parchment by means of element analyses and also to identify the type of animal that was used for the production of the parchment [231]. Metallomics analyses are also suitable for analysing paper samples, e.g., to differentiate between different types of paper [232].

### 6.3. Analysis of Exogenous Metals and Isotope Ratios

Metallomics studies can also be used to obtain information about the authors and readers of the written artefacts. For example, elevated levels of lithium could be found in books that Joseph Stalin read and that also contained comments by him. Lithium is used to treat various mental disorders. Accordingly, the authors hypothesised that the lithium residues detected were related to Stalin’s treatment of a mental disorder [32]. The same working group also analysed some pages of a book written by Johannes Kepler. Comparatively, high amounts of gold, silver, mercury and lead were detected, which is why the authors of the study suggested that Kepler may have been involved with alchemy [29]. Another study in this regard was carried out on the memoirs of Giacomo Casanova. Increased levels of mercury sulphide (cinnabar) could be detected. Mercury sulphide is highly toxic, but was previously used to treat gonorrhoea, a disease from which Casanova also suffered, which is probably why the increased cinnabar levels were detectable on the historical documents [31]. In addition, information about the writing process itself can be obtained. Metal analyses on Greek papyrus rolls were able to detect lines drawn, which the authors of the manuscripts probably used as layout templates [233].

## 7. Application of Panomics Strategies to Manuscript Research

Most of the published studies on ancient manuscripts are based on the application of a single omics strategy. However, there are also examples where different omics approaches were combined for the analysis of old manuscripts. Some examples in this context can be found in Table 3. Nevertheless, with such analyses it must always be noted that the palaeographic interpretation must not be disregarded, as was evident, for example, in the relatively well-known case of the “Gospel of Jesus’ Wife”, where the chemical analyses indicated that it was an ancient manuscript. In the meantime, however, it is assumed that it is a forgery, since the text and writing style do not correspond to the style of the time [49].

## 8. Conclusions

The use of omics technologies offers numerous possibilities for the analysis of written artefacts. Each omics strategy contributes different information, which ensures a significant increase in knowledge about the written heritage, especially through the combination of several omics disciplines. In the meantime, there are already numerous examples in which the various strategies are combined with one another, so it is possible to draw more comprehensive conclusions about the individual artefacts [12,49,151,156,190,191]. It is to be expected that this trend will continue to increase in the coming years and will also be further strengthened by the introduction of new analytical technologies and databases.

## Figures and Tables

**Table 1:** Glossary of key terms.

Term	Explanation
Archaeometry	Application of physics, chemistry, geology, and engineering sciences to analyse various archaeological materials [39].
Biocodicology	Application of genomics and proteomics approaches to ancient manuscripts [2].
Codicology	Analysis of the technical craft aspects and physical properties of a manuscript [40,41].
Omics/Paleo-omics	Comparative analysis of different elemental or molecular entireties [3]. In the context of studies on ancient manuscripts or artefacts, the focus is on the analysis of the actual object, e.g., the writing surfaces or the inks used. The prefix “paleo” means old or ancient [5,42].
Meta-omics	The focus of meta-omics studies is not the object itself, but the residues of other organism, e.g., microorganisms or the authors and readers [11]. Depending on the omics strategy chosen, the terms meta-genomics, meta-proteomics or meta-metabolomics are used, for example.
Paleography	The term refers to the study of ancient writings to be able to make spatial or temporal classifications. The focus is, for example, on the forms of the letters, spelling, or the use of typical abbreviations [40,41].
Panomics	The prefix “pan” comes from the Greek and means all, every, or whole [43]. The term panomics is used for personalised medical questions or when analyzing food. It is a networked, symbiotic approach based on genomics, transcriptomics, proteomics, and metabolomics-based data [38,44].

**Table 3:** Exemplary studies for the analysis of ancient manuscripts employing more than one omics technology.

Scientific Issue	Omics Technology	Analytical Method	Reference
Characterization of Parchment	Genomics	NGS	[55]
	Proteomics	LC-MS and MALDI-TOF	
Characterization of Parchment	Genomics	NGS	[133]
	Proteomics	MALDI-TOF	
Characterization of Parchment	Genomics	NGS	[134]
	Proteomics	MALDI-TOF	
Characterization of Parchment	Genomics	NGS	[136]
	Metallomics	FTIR, XRF, MSI	
Characterization of Parchment	Proteomics	MALDI-TOF, FTIR	[185]
	Metabolomics	Amino acid analysis, FTIR, GC-MS	
	Metallomics	FTIR, Raman, Energy dispersive X-ray spectroscopy	
Organic composition of parchment and paint binders	Proteomicse	MADLI-TOF	[190]
	Metabolomics	GC-MS	
Studying the manufacturing process of Tibetan paper	Proteomicse	LC-MS	[191]
	Metabolomics	GC-MS	
	Isotopolomics	Radiocarbon Dating	
Composition of ink binders	Proteomics	MALDI-TOF	[12]
	Metabolomics	GC-MS and LC-MS	
Analysis of paper foxing	Genomics	NGS	[151]
	Metabolomics	SALDI-TOF	
Identification of microbial communities in book collections	Genomics	Culture-dependent analysis and NGS	[152]
	Proteomics	MALDI-TOF	
Disease and treatment of nephrotic syndrome by the author Mikhail Bulgakov	Proteomics	LC-MS	[27,28,56]
	Metabolomics	GC-MS	
Infection of Casanova with gonorrhea bacteria	Proteomics	LC-MS	[31]
	Metallomics	Special mini-Hg-sensor	
Investigating the microbiological and metabolic diversity of beeswax seals	Genomics	NGS	[156]
	Metabolomics	SALDI-TOF	
Characterization and study of the biodeterioration as well as the associated microbiome of a wax seal	Genomics	Culture-dependent analysis and NGS	[155]
	Metabolomics	Raman, FTIR	
	Metallomics	Energy dispersive X-ray spectroscopy	

## Data Availability

Not applicable.
